# Communion and agency: research on social workers in China

**DOI:** 10.3389/fpsyg.2024.1293386

**Published:** 2024-12-12

**Authors:** Yangyong Zhang

**Affiliations:** Department of Social Work, School of Sociology and Anthropology, Xiamen University, Xiamen, Fujian, China

**Keywords:** communion, agency, social worker, profession, China

## Abstract

Communion and agency are two essential dimensions for understanding personality traits. This study comprised 49 in-depth interviews and three focus groups with experienced social workers in China to address the following research questions: (1) What is the orientation of Chinese social workers towards communion and agency? (2) What challenges do they encounter in their daily practice? (3) How do they interpret and utilize agency in coping with these challenges? The findings revealed three main themes: (a) Participants demonstrated a clear preference for communion over agency, reflecting a strong orientation towards social concern; (b) The professional challenges faced by participants varied significantly across career stages, with distinct requirements for agency at each stage; and (c) Participants’ understanding of agency was primarily rooted in professional competence. At different career stages, participants displayed varying levels of professional competence and agency, both of which were essential for addressing social concerns and developing strategies for long-term professional survival. The study suggests that social workers need to balance social concerns with individual development, view social work rationally as an ordinary job, and focus on enhancing both their professional competence and personal agency to achieve their aspirations.

## 1 Introduction and literature review

### 1.1 Communion and agency: key dimensions of personality

Communion and agency are two fundamental dimensions of personality (Abele and Wojciszke, 2007). Research indicates that communion is a social orientation focused on the welfare and interests of others, characterized by communal attributes. It emphasizes relationships, cooperation, interdependence, and the well-being of others (Abele and Wojciszke, 2007). The communal attributes of communion involve “strivings to integrate the self into a larger social unit” while caring for others and society (Abele and Wojciszke, 2007: p. 752; Bakan, 1966). Whether viewed from the perspective of self or others, communion is considered primary and indispensable for the survival of individuals or groups. In contrast, agency is self-oriented, focusing on self-advancement. It possesses obvious agentic attributes that “cover strivings to individuate, expand the self, and efficiently attain one’s goals” (Abele and Wojciszke, 2007: p. 752). It embodies traits related to individualism and self-development, including the pursuit of personal goals, efficiency, and independence (Abele and Wojciszke, 2007; Bartz, 2004). While agency is traditionally seen as self-centered, it can also be applied in social contexts, particularly when individuals use their personal goals to benefit others. In this way, agency incorporates both self-interest and altruism—when self-interest is fulfilled, agency can be enhanced (Abele, 2003; Abele and Wojciszke, 2007).

The literature on communion and agency spans various fields, including career development, mental health, attachment style, and reward distribution behavior (Abele, 2003; Bartz, 2004; Zhang, 2022). At the operational level, Abele et al. (2008) synthesized evidence from Belgium, Germany, Italy, Poland, and the United States to develop a set of words that characterize these two dimensions. This list includes eight positive traits and four negative traits for both communion and agency, totaling 24 words, offering a comprehensive framework for analyzing personality characteristics. These trait words have become widely recognized and provide a clear theoretical foundation for understanding the traits of social workers, particularly in the Chinese context.

This study applied Abele et al. (2008) framework, identifying 20 positive and 4 negative traits for each dimension of communion and agency (see Table 1).

**Table 1:** Characteristics of communion and agency.

	Positive characteristics	Negative characteristics
Communion	Caring	Conceited
	Helpful	Dominant, regnant
	Loyal	Egoistic, self-centered
	Polite	Hardhearted, ruthless
	Sensitive, attentive	
	Sympathetic, empathetic	
	Trustworthy, reliable	
	Understanding, considerable	
Agency	Able, competent	Insecure, uneasy
	Active	Lazy
	Assertive, firm	Shy
	Creative	Vulnerable, weak
	Independent	
	Intelligent, smart	
	Rational	
	Self-reliant	

Above table derived from Abele et al. (2008).

### 1.2 Strengths of communion and agency: key dimensions of personality

The strengths of communion and agency, as vital dimensions of personality, are evident in two primary ways. First, communion promotes social harmony and prosocial behavior. This dimension emphasizes traits that foster interpersonal relationships and social cohesion, such as empathy, warmth, trust, and cooperation. Research indicates that high levels of communion are associated with stronger social support networks and more satisfying relationships, particularly among women (Helgeson, 1994). Individuals who exhibit high communion are more likely to engage in prosocial or altruistic behaviors, such as helping others and volunteering, which positively impact both individual well-being and societal functioning (Graziano and Eisenberg, 1997). As such, communion plays a key role in community building and the development of cohesive societies, as individuals with high communion are motivated to contribute positively to their social environments. Second, agency foster personal achievement and leadership. This dimension highlights traits related to self-assertion, independence, ambition, personal achievement, and self-fulfillment. Individuals with high agency are often goal-driven, demonstrating strong motivation and perseverance to achieve personal success (Bakan, 1966). Agency is also associated with leadership qualities and the ability to influence and inspire others, such as assertiveness, confidence, and decisiveness, which are central to inspiring and influencing others (Judge et al., 2002). Thus, agency contributes to career advancement, personal success, and societal progress through innovation and leadership. Both dimensions are essential for understanding the personality characteristics of social workers. As a helping profession dedicated to social change, social work requires individuals to balance the promotion of social harmony with the pursuit of personal achievements, abilities, and leadership.

### 1.3 Social work’s social orientation and the debate on professionalization

Social work’s mission is grounded in its “social” orientation, also referred to as its “sociality.” From the early stages of professional development, the theory of “people in the situation” has underlined the dynamic changes occurring in the social environment (Chen, 2017). According to the 2014 global definition, social work is committed to social change, development, cohesion, and the empowerment and liberation of individuals. It prioritizes social equity, justice, human rights, collective responsibility, and human diversity (Li, 2015; Ornellas et al., 2018). In this sense, the social orientation of social work closely aligns with the concept of communion, which focuses on the goals of social change and the care of others and society. In China, social workers share these social goals, which include a focus on vulnerable groups, the commitment to social change, and the promotion of social equity and justice (Gan, 2017; Wang, 2020a). Furthermore, social work is a profession that requires the provision of care and empathy to service recipients (Liu, 2021; Wang, 2020a). Thus, the “social” orientation is considered foundational to the profession’s identity.

However, the debates on professionalization and social innovation in social work have persisted for over a century (Haynes, 1998). Influenced by the rise of “scientism,” the trend towards professionalization has gained significant traction, with social work scholars and practitioners increasingly advocating for professionalism and evidence-based practices. This shift has led to an emphasis on clinical service methods and techniques, often at the expense of the profession’s original “social” mission, essentially, “emphasizing professionalism over society” (Gan, 2017; Kam, 2021). Furthermore, social workers have faced criticism for abandoning their “social” mission (Guo et al., 2012), described as “unfaithful angels” (Specht and Courtney, 1995) or accused of serving as instruments for governmental social control and governance (Yin, 2011; Zhang, 2023). In response, scholars have called for social workers in China to reclaim “sociality,” reflect more critically on their practice, and explore pathways for the indigenous development of social work (Chen, 2017; Gan, 2017; Zhang, 2022).

### 1.4 The interconnection of sociality and agency in social work

While the concept of “sociality” has been widely explored, social workers’ agency also warrants attention. Scholars argue that the dimensions of “sociality” and agency in social work are not opposing forces but are, in fact, interconnected. Together, they facilitate the realization of the social work mission, values, and practices (Chen and Chen, 2017; Guo and Han, 2021; Olson, 2015). In examining social workers’ agency, professional competence is a key focus. First, competence-based education is a significant trend in international social work education, emphasizing the development of high standards of professional competence (Guo, 2021; Yuan, 2012). Second, professional competence in social work is generally recognized as encompassing three core dimensions: knowledge, skills, and values. Notably, “social” orientation is embedded within the values dimension (Damron-Rodriguez, 2008; Malmberg-Heimonen et al., 2016; Wang and Chui, 2017; Zhang et al., 2019). In the Chinese social work context, scholars define professional competence similarly, highlighting the importance of values, ethics, theoretical knowledge, and practical skills, consistent with international discussions. For example, Lei and Huang (2007) employed the Fuzzy Delphi method to construct a professional competence system, identifying 24 indicators within three dimensions: values and ethics, theoretical knowledge, and practical skills. They emphasize “pragmatic professionalism,” which refers to adapting social work practices to local, indigenous contexts. Sun and Huang (2020) introduced the concept of “flexible professionalism” as a core professional competence in China, particularly in the context of government-purchased social services. This approach involves utilizing flexible professional methods to address the diverse needs and challenges posed by government sectors, service recipients, third-party evaluators, and other stakeholders. Such flexibility serves as a positive coping strategy in daily practice. Zhang and Wang (2017) summarized ten professional capacities, including theoretical knowledge, practical abilities, learning and training skills, experience accumulation, report writing, resource linking, organizing and mobilizing skills, interpersonal communication, artistic performance, and psychological resilience. Additionally, Wang and Chui (2017) developed and validated the Perceived Social Work Competence Scale (PSWCS) to assess social work students’ self-evaluation of their competence across ten subscales in two main categories: meta-competence and procedural competence. These subscales include professional knowledge development, teamwork, relationship building, professional resilience, therapeutic skills, insight, supportive skills, case management, and community work. Despite these advancements, there remains a lack of comprehensive exploration of professional competence requirements across different professional stages and service fields. Further research is needed to address these gaps and provide a more nuanced understanding of the evolving nature of professional competence in social work.

Chinese scholars have increasingly highlighted the pressing need to address the professional competence of social workers in China. Zhu and Chen (2013), in their case study of the Guangzhou Family Comprehensive Service Center, found that the work effectiveness of social workers fell short compared to local community workers. Other studies have also identified inadequate professional competence as a key obstacle to the development of social work in China (Zhang and Wang, 2017). Several factors contribute to this issue. First, there is a mismatch between the supply of social work professionals and the growing demand for social services. The low entry barriers to the profession, including national professional qualification exam, have led to the entry of individuals from diverse, non-professional backgrounds, resulting in varying levels of professional competence. Second, despite its rapid development, social work in China remains in its early stages of professionalization. This delayed development means that social workers face challenges in accumulating indigenous practical experience and building the necessary professional competence (Zhang, 2022, 2023). Additionally, the low social recognition of social work and the complexity of indigenous social work contexts require social workers to develop multidimensional professional competence to navigate their roles effectively (Tong, 2019; Zheng, 2020).

*Hypothesis 1:* Chinese social workers demonstrate significantly higher levels of communion than agency.

Concerns have been raised that the social work profession currently tends to prioritize professionalism over its social orientation, with the latter being questioned, downplayed, or even marginalized (Gan, 2017; Guo et al., 2012). Therefore, it is crucial to revisit the profession’s two dimensions of communion and agency. First, exploring Chinese social workers’ orientation towards these dimensions will help clarify the social orientation of the profession. Second, addressing concerns regarding social workers’ agency and professional competence is crucial for understanding their engagement with these concepts.

Studies consistently show gender differences in communion and agency. Women are often socialized to exhibit more communal traits, while men are encouraged to display more agentic traits (Eagly and Wood, 2012). In China’s social work profession, women comprise a significant majority, accounting for 79.04% of the workforce (Yuan et al., 2021). This aligns female social workers with the communion dimension and the social mission of social work as a helping profession, often leading to greater investment in emotional labor (Guo and Han, 2021). In contrast, male social workers are more likely to emphasize rationality, logical thinking, and management skills, reflecting a strong orientation towards agency (Sun and Zhou, 2019). This gender-based differences have important implications for understanding gender roles within the Chinese social work profession.

Cultural contexts also shape the expression and value placed on communion and agency. In collectivist cultures, such as China’s, communal traits are emphasized, while individualistic cultures prioritize agency (Triandis, 1995). Research also suggests that Chinese social workers’ decision to enter the profession reflects collectivist values such as patriotism, dedication, friendship, and a strong commitment to the social work mission. These values drive social workers to contribute to society and engage with their communities (Wang et al., 2021; Zhang, 2022, 2023).

*Hypothesis 2:* Female social workers will demonstrate significantly higher levels of communion than male social workers, while male social workers will exhibit significantly higher levels of agency.

### 1.5 Research gaps and aims

This study identifies significant research gaps concerning the personality traits of communion and agency within the field of social work. Social workers, as distinct from the general population, possess unique professional skills, values, and ethics aimed to fostering individual, group, community, or societal change. However, existing research has largely overlooked the exploration of communion and agency traits within the helping professions, particularly among social service practitioners.

To address these gaps, this study aims to explore the orientation towards communion and agency among Chinese social workers. In-depth interviews will be conducted with experienced practitioners in three Chinese cities, Shanghai, Shenzhen, and Xiamen, focusing on their career experiences in social work. The study will also examine the professional challenges these social workers face and explore their interpretations and applications of agency at different stages of their careers.

## 2 Research methods

### 2.1 Research data and methodology

The data for this study were sourced from a database established by a National Social Science Fund project in China. This database comprises in-depth interviews with 49 experienced, licensed social workers and three focus group interviews conducted in three cities: Shanghai, Shenzhen, and Xiamen. These cities were selected to ensure a diverse sample of participants, achieve data saturation, and maintain feasibility for data collection. The study was undertaken between 2018 and 2020, resulting in verbatim transcripts exceeding 1.5 million words.

### 2.2 Participant criteria

The study participants met the following criteria: (1) licensed social workers who had completed university-level social work education and held a social work diploma; (2) individuals working in a social work service organization (SWSO) or a non-profit organization (NPO) for more than 1 year; and (3) social workers with over 5 years professional experience intending to remain in the profession long-term. Exclusions included social workers who qualified solely through the national licensure examination without a social work diploma or those worked in government or community neighborhood committees (CNC), as their roles were primarily administrative.

### 2.3 Data collection and ethical considerations

Data collection took place in two phases. Phase One included in-depth interviews, while Phase Two consisted of three focus groups. All interviews and focus groups were conducted in person by the author. To recruit participants, the author employed both professional networks and a snowball sampling technique. Four individuals were designated as gatekeepers in Shenzhen and Shanghai, leveraging their networks to identify eligible participants. Data saturation was confirmed after 49 individual interviews.

During Phase One, in-depth interviews revolved around 14 core questions, such as: “What difficulties and challenges do you face regularly in your work?” and “What coping strategies do you utilize in your daily life and throughout your career?” These interviews explored the professional challenges participants faced, their coping strategies, and the evolution of their entire career journey from tertiary education onwards. Forty-three participants were interviewed once, while six were interviewed twice due to the emergence of further meaningful insights after the first interview. Each interview lasted approximately 2 hours. Demographic information was collected during this phase as well. In Phase Two, participants were invited to participate in three parallel focus groups held in each city. The purpose of the focus groups was to present the preliminary findings, gather feedback, and identify new ideas or themes.

All participants provided informed consent for their involvement in interviews and focus groups. All sessions were audio-recorded and transcribed verbatim. The study received ethical approval from the Human Subjects Ethics Committee of the author’s affiliated university.

### 2.4 Data analysis

Two approaches were utilized for data analysis. First, Python software was applied to conduct a word frequency analysis using Abele et al. (2008) list of characteristic words related to communion and agency (see Table 1). This analysis helped identify participants’ orientation towards these two dimensions by examining the frequency of relevant words in the transcripts. Discourse scenarios reflecting communion and agency were then extracted. Second, NVivo 12 Plus software was used to code all transcripts from the interviews and focus groups, facilitating the identification of the professional challenges faced by the participants and their interpretation of personal agency.

### 2.5 Participant demographics

Among the 49 participants, there were 41 females and 8 males. Of them, 31 were married, while 18 were unmarried. The average age of participants was 32.7 years, with an average of 8.6 years of work experience. Participants were distributed across three cities was as follows: 15 from Shanghai (coded SH01 to SH15), 16 from Shenzhen (coded SZ01 to SZ16), and 18 from Xiamen (coded XM01 to XM18). Participants were categorized based on their professional roles: 22 were organizational managers (OM, senior level), including directors and deputy directors; 25 were program officers or managers (PM, middle level), including supervisors, program or departmental directors, regional managers, or service center directors; and 2 were frontline social workers (FSW, junior level). All OMs had previous experience as FSWs and PMs, and all PMs had prior FSW experience. The participants’ work encompassed various areas, such as elderly care, children’s services, community services, and community governance.

## 3 Findings

The study findings are organized into three key areas: (1) analysis of participants’ orientation towards communion and agency; (2) identification of the professional challenges participants faced at different career stages; and (3) participants’ interpretation of personal agency.

### 3.1 Orientation towards communion and agency

To analyze participants’ orientation towards communion and agency, word frequency analysis was conducted on the transcripts of the 49 in-depth interviews using Python software. The analysis focused on the positive characteristic words related to communion and agency, as outlined in Table 1. As shown in Table 2, participants exhibited a stronger orientation towards communion than agency [frequency_communion = 0.70, frequency_agency = 0.39 (*p* < 0.01)]. These results indicate that Chinese social workers, as professionals dedicated to helping others, prioritize caring for others and promoting social well-being. Therefore, Hypothesis 1, which posits that Chinese social workers’ communion would demonstrate significantly higher levels of communion than agency, was supported.

**Table 2:** Positive word frequency statistics of communion and agency.

Sample	Agency	Communion	Frequency_Agency	Frequency_Communion	Sample	Agency	Communion	Frequency_Agency	Frequency_Communion
SH01	38	23	0.50	0.30	SZ11	21	33	0.34	0.53
SH02	26	40	0.37	0.58	SZ12	22	13	0.56	0.33
SH03	28	25	0.81	0.72	SZ13	4	22	0.15	0.80
SH04	30	45	0.65	0.98	SZ14	21	10	0.53	0.25
SH05	27	40	0.61	0.91	SZ15	11	20	0.42	0.77
SH06	15	38	0.35	0.88	SZ16	9	20	0.17	0.38
SH07	33	48	0.64	0.93	XM01	29	74	0.39	1.01
SH08	32	25	0.59	0.46	XM02	20	26	0.45	0.58
SH09	25	51	0.39	0.79	XM03	5	26	0.14	0.73
SH10	22	16	0.43	0.32	XM04	15	38	0.30	0.76
SH11	13	8	0.32	0.20	XM05	7	26	0.15	0.57
SH12	23	60	0.42	1.09	XM06	14	36	0.30	0.77
SH13	15	28	0.39	0.73	XM07	20	43	0.44	0.94
SH14	13	15	0.34	0.39	XM08	4	20	0.16	0.78
SH15	33	28	0.65	0.55	XM09	21	33	0.42	0.66
SZ01	43	76	0.69	1.22	XM10	33	14	0.50	0.21
SZ02	10	28	0.23	0.64	XM11	31	45	0.53	0.77
SZ03	10	43	0.27	1.17	XM12	24	49	0.35	0.72
SZ04	18	35	0.38	0.74	XM13	23	51	0.33	0.73
SZ05	8	38	0.20	0.94	XM14	10	23	0.31	0.70
SZ06	14	22	0.30	0.46	XM15	21	56	0.43	1.15
SZ07	21	49	0.33	0.76	XM16	27	48	0.38	0.68
SZ08	13	21	0.40	0.64	XM17	13	35	0.35	0.94
SZ09	9	28	0.24	0.74	XM18	21	50	0.28	0.68
SZ10	18	44	0.33	0.82					
**Average**						19.65	34.39	0.39**	0.70**

** *p* < 0.01.

Paired sample *t* test results (see Table 3) showed that participants’ communion (M = 0.70, SD = 0.25) was significantly higher than their agency (M = 0.39, SD = 0.15), *t* = −7.58, *p* < 0.001. These results support Hypothesis 1, reinforcing that Chinese social workers prioritize communion over agency. Additionally, a significant positive correlation was found between a participant’s age and their duration of employment in social work (r = 0.86, *p* < 0.01). However, no significant correlations were observed between other variables, and there was no evidence of an accumulation effect of age and work experience on participants’ levels of communion and agency. This contrasts with existing literature; for example, Katz et al. (2024) suggest that child protection workers, including social workers, experience improved resilience and mental health with increased work experience. The discrepancy may be attributed to factors such as the small sample size in this study and the relatively homogeneous work experience among participants (all>5 years, and M = 8.6 years). Future research could address these limitations by employing larger samples and conducting quantitative analyses.

**Table 3:** Correlation analysis of variables.

	*M*	SD	1	2	3	4	5	6
1: Agency	0.39	0.15						
2: Communion	0.70	0.25	0.03					
3: Gender	N/A	N/A	0.03	−0.26				
4: Age	32.69	4.60	0.18	−0.23	−0.12			
5: Education	N/A	N/A	0.24	0.01	0.03	0.25		
6: Career stage	N/A	N/A	0.02	−0.15	0.26	0.21	0.27	
7: Years of work	8.63	2.86	0.26	−0.20	−0.18	0.86**	0.22	0.26

** *p* < 0.01. N/A, not applicable. Gender: 0 = female, 1 = male. Education: 0 = below bachelor, 1 = bachelor, 2 = master and above. Career stage: 0 = FSW, 1 = PM, 2 = OM.

**Figure 1:**
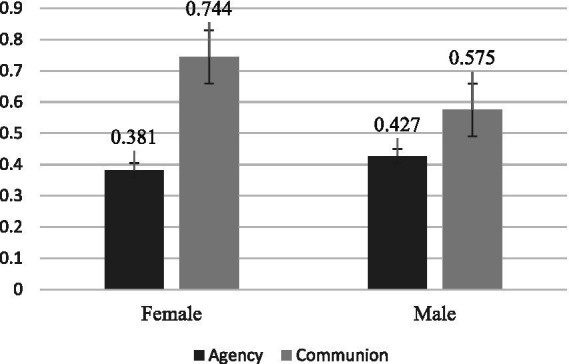
Agency and communion by gender.

A mixed 2 (agency vs. communion) *2 (male vs. female) ANOVA was conducted with age, education, career stage, and years of work as covariates. The results (see Figure 1) showed a significant gender difference in participants’ agency (*F*(1.43) = 4.91, *p* = 0.032, *η_p_^2^* = 0.10), with male participants scoring higher than female participants. This finding supports Hypothesis 2. Additionally, female participants showed a significantly higher level of communion than agency (*p* < 0.001), partially supporting Hypothesis 2 by indicating a significant gender difference in participants’ agency, with female participants exhibiting stronger communal traits than agentic ones. However, this gender difference was not observed among male participants (*p* = 0.082). This may be attributed to the small number of male participants (*n* = 8). These findings align with previous research, which suggests that male social workers emphasize rationality, logical thinking, and management skills more than their female counterparts, thus prioritizing agency over communion (Sun and Zhou, 2019). Furthermore, in the Chinese context, social work professionals are often influenced by collectivist culture (Wang et al., 2021) and are required to invest significant emotional labor (Guo and Han, 2021). This cultural influence may lead female social workers to prefer the profession, thereby exhibiting distinct communal traits (Zhang, 2022, 2023).

A similar mixed [2 (agency vs. communion)] *3 (FSW vs. PM vs. OM) ANOVA was conducted with age, education, career stage, and years of work as covariates. The results indicated no significant differences between participants’ agency and communion across different career stages (*F*(2.42) = 1.76, *p* = 0.184, *η_p_^2^* = 0.08).

### 3.2 Professional challenges across career stages

Based on the word frequency analysis, in-depth interview transcripts were matched with characteristic words related to communion and agency. Subsequently, NVivo 12 Plus software was used for further coding and thematic analysis of the interview and focus group data. Finally, using Bourdieu’s practice theory as a theoretical framework (Bourdieu, 1977; Bourdieu and Wacquant, 1992), the study identified the professional challenges participants faced at different career stages (see Table 4) and their interpretations of personal agency (see Table 5).

**Table 4:** Challenges faced by social workers.

Challenges faced by social workers	No. of participants	No. of references	Typical quotations
	(*N* = 49)	421	
**1. Challenges of FSWs**	42	155	SZ15: In the first 2 or 3 years, it was a process of vocational adaptation…Social work is a new profession; neither service recipients nor the sponsors were ready…each stakeholder was learning from each other. For me, it was a process of accumulation [of work experience]. what should I do and how could I do it? At that time, I had no idea and was confused.
1) Challenges resulting from previous inadequate social work education: gap between inadequate social work education and demand for professional methods in daily practice.	16	28	
2) Challenges in daily practice: shortage of direct service skills; difficulties of adaptation in social work fields.	29	86	
3) Frequent turnover intention: low professional recognition, low starting salary, etc.	20	41	
**2. Challenges of PMs**	37	150	SH04: In the first year I became a PM, I was really exhausted. I clearly saw shortages and challenges rushing towards me. Everyone [other social workers] had different experiences…The difficulty of managing a project and a team made the first year of PM very tough. That year was particularly difficult for me.
1) Role transition pressure: from FSW to PM	24	42	
2) Project administrative work overload	22	42	
3) Inadequate project management capacity and experience	11	18	
4) Confusion about personal career plans	20	48	
**3. Challenges of OMs**	8	15	XM02: After clarifying the mission of our organization…I communicated with the other two founders, and planned to develop pure social work services matching its mission…But, we had to get a project funded firstly, the organizational economic pressure was very high…We almost did not make it when we did the second project because we did not grasp the policy well, and many resources and relationships with stakeholders.
1) Frustrated role transition: roles, high capacity and responsibility challenges	3	3	
2) The pressure of organizational governance and sustainable development	4	6	
3) The temptation of higher salaried jobs in other professions	5	6	
**4. Common challenges for all**	36	101	
1) A large caseload, burnout and stress	19	31	
2) A comparatively low income	25	46	
3) Social work values and ethical conflicts	12	24	

**Table 5:** Social workers’ interpretation of agency.

Agency	No. of participants	No. of references	Typical quotations
	(*N* = 49)	264	
**1. Professional competence in direct services**	33	111	XM03: It will be more beneficial for social workers to understand the feelings and situation of clients first, and then establish a relationship…this process will be of great help in supervision. I have also accumulated some experiences and explore methods previously [that]…will be more grounded and responsive to the needs of [clients] in their real situations.
1) Service skills: case work, group work, activity organization and implementation, needs assessment, listening, resource integration, etc.	29	48	
2) Supervision skills	20	28	
3) Social work values and ethics	25	35	
**2. Project and team management capacities**	30	56	SH06: As a manager…I should have the right attitude. I also need to use some methods because everyone has different motivations and ideas…Professional and personal charisma [need to be demonstrated]…In social work organizations, understanding and respect in social work are important. If I only communicate and give feedback based on tasks, it seems to conflict with the values of social work.
Project management; team management; time management; file management; project research and development; and research capabilities			
**3. Interpersonal communication skills**	29	40	SH06: The first is adaptation skill, which satisfies the needs of different scenarios. The second is stress resistance skill when facing setbacks…as well as learning the ability to respond to different services and needs…in order to present continuous innovations. Emotional intelligence is also important…dealing with different internal and external relationships, I think most of it is about communication.
Communication ability; expression ability; coordination and relationship management skills			
**4. Learning and adaptation ability**	27	42	
Learning and environment adaptation ability; stress resistance ability; psychological adjustment and emotional management ability; open attitude, etc.			
**5. Organizational management capacities**	10	15	SH09: I think it is necessary to have a clear understanding and recognition of the direction of organizational development and strategic development, and then to pass it on to team members well. This is to ensure they are applied correctly…and maintain the cohesion of the organization. In addition, the organization must have its values and carry out continuous advocacy to ensure social workers internalize values and externalize them to improve their practice.
Leadership; organizational management; financial planning and development; communication and marketing; innovation ability, etc.			

As shown in Table 4, several key findings emerged from the analysis of professional challenges. First, while social workers expressed a strong desire to care for others and society, they encountered common challenges in their daily practice, including heavy workloads, burnout, economic pressure, low income, and conflicts of values and ethics. Participant SZ04 provided a vivid example:

When I worked as a frontline social worker in a public hospital in Shenzhen, besides the heavy workload, lack of experience, and low income, I also felt skepticism from doctors, nurses, patients, and hospital leadership. They even disparaged social work and refused to cooperate with me (SZ04: 31-year-old female PM in Shenzhen).

Second, the professional challenges faced by social workers varied across career stages, reflecting different roles and distinct requirements for agency at each stage, as well as varying professional competencies. For example, FSWs primarily focused on direct service skills, while PMs needed to master program management and define professional boundaries. OMs, on the other hand, were responsible for organizational governance, program development, and community engagement. Participant XM01 described the challenges encountered as he progressed from PM to OM:

When I first became a program manager, I didn’t know how to manage social service programs or lead a team. Two years later, I was assigned to manage two more programs and encountered frequent resignations among social workers. Later, when I took on organization management responsibilities, I faced difficulties such as creating unique programs and brands, limited resources, intense competition between organizations, and significant pressure for the organizational survival (XM01: 32-year-old male OM in Xiamen).

These findings underscore the evolving and multifaceted nature of the challenges social workers face throughout their careers. They also highlight the importance of both communion and personal agency in navigating these challenges.

### 3.3 Personal agency in social work practice

Table 5 presents a multidimensional analysis of personal agency among participants. First, personal agency was primarily interpreted based on professional competence, encompassing direct service skills, supervision skills, and professional values and ethics. For example, participant SH06 noted:

As a social worker, it’s essential to have basic professional skills, service abilities, values, and ethics. These form the foundation for delivering professional social services. Additionally, we need the ability to supervise young social workers, manage programs, and lead teams (SH06: 31-year-old female PM in Shanghai).

Second, the analysis also revealed that as social workers advanced in their careers, particularly in PM and OM roles, they increasingly emphasized the importance of program management, leadership, and organizational management skills. Participant SZ01 reflected on the evolving skill sets required at each stage of her career:

Moving from a frontline social worker to a program manager, and now to an organizational manager, I have noticed distinct differences in the required skills at each stage. Frontline social workers need to master micro-level direct service skills, while later on, there is an increasing need to acquire abilities in program management, leadership, organizational management, policy practice, and so on (SZ10: 36-year-old female OM in Shenzhen).

Third, interpersonal communication skills and the ability to learn and adapt were widely recognized by the participants as crucial elements of personal agency. These skills were especially important for managing professional challenges and supporting social workers’ engagement with the community, including communication, coordination and relationship management, learning ability, and environmental adaptation and adjustment ability. Participant SH09 provided an example of how communication and adaptation were integral to her work:

I have always emphasized communication with leaders of government sectors at different levels and maintained good cooperative relationships with them. The process of communication and interaction with them is also a continuous learning and adaptation to the government discourse system. I believe these are essential basic skills that social workers must possess (SH09: 34-year-old female OM in Shanghai).

These findings highlight the crucial role of both communion and personal agency in navigating the complex and evolving challenges faced by social workers throughout their careers. The development of personal agency, particularly through the acquisition of professional competence and interpersonal skills, plays a critical role in enabling social workers to overcome challenges and contribute to the community effectively.

## 4 Discussion and conclusion

### 4.1 Social concerns: social workers’ mission and method

This study reveals that social workers in China are primarily oriented towards caring for others and society, which aligns with the core mission and values of the social work profession. Social concern is a fundamental goal and mission of social work, emphasizing the service of people over the pursuit of personal gain. Participants in this study adhered to this mission by responding to the needs of service recipients, particularly vulnerable groups, addressing social problems, promoting social change, and contributing to the development of the social governance community. This community includes the government, SWSOs, CNCs, social workers, enterprises, volunteers, and other stakeholders (Gan, 2017; Wang, 2020a).

In practice, social workers in this study emphasized that caring for society is not just a theoretical value but also a core method in social work services. They analyzed the needs and problems of service recipients within specific community contexts, rather than focusing solely on individual or familial concerns. Social workers considered social problems from a broader perspective, seeking solutions that meet the diverse needs of service recipients while utilizing available social resources to ensure comprehensive care (Chen, 2017; Zhang, 2022).

### 4.2 Communion: a survival strategy for social workers

The study highlights communion as a coping strategy that enables social workers to maintain long-term career survival. Participants reported encountering numerous professional challenges, including low social recognition, high work pressure, and a lack of professional competence and experience, challenges that echo issues identified in existing literature (Xu, 2017; Zeng et al., 2020; Zhang, 2022, 2023). Participants viewed communion as a vital mechanism for obtaining social support, accessing resources, and gaining recognition within their professional context, which in turn promoted the social status of the social work profession. Additionally, participants emphasized the importance of participating in capacity-building activities such as training and supervisor-fostering schemes, organized by the government, universities, social work associations, and other stakeholders. These efforts were designed to enhance their personal competence and elevate the social status of social workers (Zhang, 2022, 2023).

Theoretically, this study extends the concept of communion identified in the general public, building on the work of Abele and Wojciszke (2007), Abele et al. (2008), and Bartz (2004), who explored the benefits of communion as a dimension of personality traits. For social workers, however, communion is not just a personality trait but an internalized element of their professional habitus—a construct shaped by social work education and professional experience (Bourdieu, 1977; Bourdieu and Wacquant, 1992). Thus, communion serves not only as an intrinsic personality trait but also as a professional method and survival strategy in the career of social workers.

### 4.3 Agency: professional competence in social concerns

Participants in this study utilized their agency to address social concerns, reflecting the importance of their professional competence. The findings expand the traditional understanding of social work as a profession that extends beyond offering compassion and care. For social workers, communion and agency are intertwined, as agency involves the application of professional skills and knowledge in service of social concerns. Literature on the core professional competencies of social workers in China (Lei and Huang, 2007; Sun and Huang, 2020; Zhang and Wang, 2017) suggests that: First, social workers should adhere to professional values and ethics to provide care and services that benefit others and society. Additionally, they should cooperate with service recipients, sponsors, partners, volunteers, and other stakeholders to jointly draw a social blueprint and address social issues, rather than seeking personal gain. Second, social workers are expected to possess theoretical knowledge and analytical skills to address social problems from an appropriate theoretical perspective. Third, they should also apply specific service methods and skills, such as case work (focused on individual concerns), group work (focused on group dynamics), community work (addressing community issues), and social administration (concerned with macro-level social issues and inter-organizational relationships), to serve others and society to achieve the mission and goal of social concerns. This study supports these viewpoints and further emphasizes the importance of diverse service methods in achieving the goals of social concern. Key skills such as empathy, listening, resource linkage, service users’ self-determination, and value neutrality were also highlighted as essential for effective service delivery. Furthermore, participants noted the importance of program and team management, organizational development and management, interpersonal communication, and learning and adaptation.

However, participants also acknowledged areas where their professional competence could be enhanced, which resonates with findings in previous research (Zhang, 2022, 2023). These areas for improvement included:

***Understanding indigenous social work context***: Participants stressed the importance of gaining a deeper understanding of the indigenous social work context, including the development of social work from an embedded model to an integrated one (Wang, 2020b). They also continuously updated knowledge about policies, government initiatives, and community development.
***Refining indigenous social work theory and service models***: Social workers expressed the need to invest more effort into developing and refining indigenous social work theories and service models. Examples include models such as the embedded development model and the linkage model involving multiple stakeholders (Zhang, 2022).
***Improving professional competence of localized service methods***: There was also a call for the development of localized service methods tailored to specific service groups. This involves developing specific skills and strategies that address the unique needs and challenges faced by various populations within the local context.

From an agency perspective, this study borrows Abele and Wojciszke (2007) and Abele et al. (2008) framework to interpret social workers’ agency. For social workers, agency is not just a personality trait, but also a reflection of professional competence, which includes management capacity, communication abilities, and learning and adaptation ability. These multidimensional competencies are essential for providing effective services, solve social problems, promoting social change, maintaining stability in social work fields and their sub-fields, and fostering cooperation among stakeholders and social actors (Fligstein and McAdam, 2011, 2012).

### 4.4 Balancing “society” and “individuality”

While participants demonstrated the mission of social work as a profession oriented towards social concern, they also identified challenges in balancing social responsibility with personal and professional growth. The entry threshold, professionalism, and unique features of the social work profession in China remain relatively low, and as the profession matures, it is essential to gradually raise the qualifications for becoming a licensed social worker to reflect its growing social responsibilities.

Social workers must prioritize their professional competence to improve their social status and recognition, while also maintaining a value orientation towards social concerns. This process will help realize the profession’s aspirations and improve its public image (Boddy et al., 2018). Moreover, social workers should be treated as ordinary professionals with career aspirations similar to those in other fields (Koenig and Spano, 2007; Luo and Chui, 2020). As part of this process, social workers should not be content with low salaries that are “relatively unresponsive to its price” (Barth, 2003: p. 17), and must advocate for better compensation, career growth, and higher social status to promote the formation of a high-level, professional social work workforce.

Attention to self-care is also crucial, as social workers often face significant pressures in their roles, leading to negative emotions, burnout, and the intentions to leave the profession (Zeng et al., 2020; Zhang et al., 2014; Zhang, 2023). While serving others and society and addressing social problems, social workers need to recognize the importance of self-care, supported by emotional support from families, colleagues and supervisors, professional supervision, social recognition, and self-adjustment skills. Social workers should also prioritize work-life balance and emotional well-being to enhance their ability to continue serving others effectively.

### 4.5 Conclusions and contributions

#### 4.5.1 Conclusion

This study highlights the centrality of social concern to the mission of social work in China, demonstrating that, while the professional mission of social work emphasizes a “social” orientation, there is a growing trend towards emphasizing professionalism over societal focus in the development of social work in China. Through an in-depth exploration of Chinese social workers’ communion and agency, this study provides empirical evidence of the profession’s dedication to its social mission while also reshaping its “social” image.

The study examined the two essential personality traits of communion and agency and investigated the professional challenges Chinese social workers faced at different career stages. The findings showed that Chinese social workers prioritize communion over agency, particularly among female professionals, underscoring their strong orientation towards social concerns. Additionally, a significant gender difference in agency was observed, with male social workers exhibiting higher levels of agency than their female counterparts.

The professional challenges faced by Chinese social workers at different career stages revealed notable differences in the requirements for agency, which were linked to role evolution and shifting professional competence. Participants’ interpretations of agency were primarily based on their professional competence. At different career stages, participants distinguished between professional competence and the utilization of their agency, which was employed as both a tool to fulfill their social mission and as a coping strategy to ensure long-term professional survival.

The study suggests that Chinese social workers need to find a balance between their societal mission and individual aspirations, prioritizing the improvement of professional competence while also pursuing both personal and professional goals.

#### 4.5.2 Contributions

This study makes several key contributions:

***Theoretical and practical insights into personality traits***: By examining the personality traits of Chinese social workers along the dimensions of communion and agency, this study underscores the profession’s social rather than profit-oriented nature, highlighting its foundational “social” orientation. This contributes to understanding how social workers define their professional identities in China.
***Empirical understanding of personal agency***: The study provides an in-depth interpretation of how Chinese social workers, particularly in the emerging social work context, understand and define personal agency. It also reveals significant differences in these interpretations across different career stages, providing valuable insights into how agency evolves as social workers advance in their careers.
***Balancing professional and personal aspirations***: The study emphasizes the need for Chinese social workers to not only adhere to their social mission but also to focus on improving their capacities and professional competence. It suggests that social workers should pursue both their personal and professional aspirations to strengthen their career development and contribute meaningfully to the social work profession.

### 4.6 Limitations and future research

The study drew on the accounts of 49 selected social workers from three cities and analyzed data from in-depth interviews. All participants had received professional social work education, held a college-level or higher diploma, worked in SWSOs, and had more than 5 years of experience in the social work field. While these criteria facilitated a focus on experienced social workers with professional training, they also restricted the diversity of the sample, which may limit the generalizability of the findings.

Future research could expand the sample to cover social workers from a wider range of geographical areas, including provinces, cities, and districts where social work is at different stages of development. It could also focus on licensed social workers working in government sectors and CNCs, or non-licensed social workers in SWSOs, conducting comparative research on related topics to provide a broader understanding of the experiences and challenges faced by different groups within the profession.

## Data Availability

The raw data supporting the conclusions of this article will be made available by the author, without undue reservation.
